# Platelet-to-lymphocyte ratio as a potential marker for routine management of renal anemia in maintenance hemodialysis patients: A single-center observational study

**DOI:** 10.1097/MD.0000000000047075

**Published:** 2026-01-09

**Authors:** Tingting Yang, Jiling Zhang, Tianqing Cao, Chengeng Liu

**Affiliations:** a Department of Clinical Laboratory, Beijing Shunyi District Hospital, Beijing, China; b Department of Neurosurgery, Beijing Tiantan Hospital, Capital Medical University, Beijing, China; c The National Clinical Research Center for Mental Disorders and Beijing Key Laboratory of Mental Disorders, Beijing Anding Hospital, Capital Medical University, Beijing, China.

**Keywords:** correlation, maintenance hemodialysis, Novel inflammatory markers, renal anemia

## Abstract

Renal anemia is a common complication in maintenance hemodialysis (MHD) patients, closely linked to higher cardiovascular event risk, reduced quality of life, and poor prognosis. This study explored the correlation between novel peripheral blood inflammatory markers and renal anemia in MHD patients. A total of 142 regular MHD patients (January 2022–June 2023) were divided into renal anemia group (Hb < 110 g/L, n = 75) and non-anemia group (Hb ≥ 110 g/L, n = 67) per 2017 Kidney disease guidelines; 74 healthy controls were included. Patients with hematologic disorders, recent acute blood loss, etc, were excluded. neutrophil-to-lymphocyte ratio, monocyte-to-lymphocyte ratio, platelet-to-lymphocyte ratio (PLR), and systemic immune-inflammation index (SII) were measured/calculated. Age and gender were comparable across groups (*P* >.05). Mann–Whitney *U* test, Pearson correlation, and receiver operating characteristic curve analysis were used. neutrophil-to-lymphocyte ratio, monocyte-to-lymphocyte ratio, PLR, SII were higher in both MHD groups than controls; PLR and SII were higher in anemia group than non-anemia group. All 4 markers correlated negatively with Hb. PLR and SII had diagnostic value for MHD renal anemia, with PLR being optimal (AUC = 0.844, 95% CI: 0.774–0.899, *P* < .001; sensitivity = 76.0%, 95% CI: 65.2%–84.3%;specificity = 85.1%, 95% CI: 74.7%–91.7%;optimal cutoff = 143.4). PLR is associated with renal anemia in MHD patients, potentially serving as an accessible screening marker. However, single-center limitation requires validation in large-scale multi-center studies before clinical promotion.

## 1. Introduction

Hemodialysis is the key means to prolong the survival of patients with chronic renal failure.^[1]^ With the continuous progress of blood purification technology, the living conditions of patients with maintenance hemodialysis (MHD) have improved. However, with the extension of dialysis time, patients may have a variety of complications, which seriously affect their quality of life and prognosis.^[2]^ Among them, renal anemia is one of the common complications of MHD patients,^[3]^ and its incidence rate gradually increases with the aggravation of renal function damage. According to statistics, as many as 90% of dialysis patients will have renal anemia.^[4]^ A large number of evidence-based medical data show that anemia is a risk factor for poor prognosis of MHD patients and the main reason for high hospitalization rate and high mortality rate of MHD patients.^[5]^ Therefore, improving anemia is of great significance to the prognosis of MHD patients.

Studies have shown that the pathogenesis of renal anemia is very complicated. In addition to the relative lack of erythropoietin, secondary hyperparathyroidism, shortened life span of red blood cells, malnutrition and other factors, persistent inflammation also plays an important role in the pathogenesis of renal anemia.^[6,7]^ Inflammation mainly induces anemia through the following mechanisms: Inflammatory cells increase the expression of hepcidin through various channels and aggravate anemia.^[8]^ Affect the production of erythropoietin (EPO): Under the stimulation of inflammation, monocytes and macrophages produce a variety of cytokines, which affect the growth of erythroid precursor cells, resulting in the reduction of EPO production.^[9]^ Shorten the life span of red blood cells: Macrophages can be activated in the inflammatory state. Macrophages can not only remove the cell membrane of aging red blood cells, but also increase the coating of immunoglobulin or immune complex on the surface of red blood cells, resulting in excessive removal of red blood cells, which in turn leads to a decrease in the number of red blood cells in the body.^[10]^ Therefore, it is of great value to find inflammation early and take corresponding measures to improve anemia.^[11]^

At present, commonly used inflammation detection indicators include C-reactive protein (CRP), interleukin -6 (IL-6) and tumor necrosis factor –α (TNF-α). However, these inflammatory indicators are difficult to be routinely detected in MHD patients due to methods or cost problems. Therefore, an economical and effective index is needed to detect the inflammatory state of patients.

In recent years, new inflammatory indicators derived from peripheral blood cell analysis, such as neutrophil-lymphocyte ratio (NLR), monocyte-lymphocyte ratio (MLR), platelet-lymphocyte ratio (PLR) and systemic immune inflammatory index (SII), have attracted much attention because of their convenience, repeatability and low-cost, and have become prognostic markers of various chronic diseases such as cardiovascular diseases and tumors.^[12,13]^ However, there are relatively few studies on the relationship between these new inflammatory indicators and MHD renal anemia.

We hypothesized that PLR and related inflammatory indices would be associated with anemia severity and might have diagnostic value in MHD patients. To test this hypothesis, we enrolled 142 MHD patients who received regular dialysis in our center from January 2022 to June 2023, and analyzed the relationship between NLR, MLR, PLR, SII levels and renal anemia, aiming to provide evidence for optimizing routine anemia management in MHD patients.

## 2. Methods

### 2.1. General data

A total of 142 patients undergoing regular maintenance hemodialysis at our hospital’s dialysis center from January 2022 to June 2023 were selected as study subjects. According to the 2017 Kidney Disease Guidelines,^[14]^ patients were divided into 2 groups: the anemia group (hemoglobin (Hb) < 110 g/L) and the non-anemia group (Hb ≥ 110 g/L). The anemia group consisted of 75 patients (44 males and 31 females), aged 31 to 79 years, with an average age of 58.95 ± 11.84 years. The non-anemia group comprised 67 patients (38 males and 29 females), aged 34 to 81 years, with an average age of 60.32 ± 11.54 years. Both groups underwent regular dialysis, with a frequency of 2 to 3 times per week, each session lasting 4 hours. Exclusion criteria were as follows: patients with a history of hematologic disorders; patients who had experienced gastrointestinal bleeding or other acute blood loss within the past 6 months; patients with a history of blood transfusion within the past 3 months; patients with severe infections, heart failure, or malignant tumors; patients with a history of trauma or surgery within the past 3 months; patients who had been on dialysis for less than 3 months, had inadequate dialysis, or had insufficient erythropoietin dosage. Additionally, 74 relatively healthy patients who visited our hospital’s health examination center were selected as the healthy control group, including 42 males and 32 females, aged 34 to 80 years, with an average age of 60.07 ± 10.58 years. This group had no recent acute or chronic infections, no cardiovascular, neurological, or renal diseases, and had not taken any anti-inflammatory medications or other drugs that could affect the observed parameters. There is no statistical difference in age and gender among the 3 groups (*P* > .05), indicating comparability. Written informed consent was obtained from all participants or their guardians, and the study was approved by the Ethics Committee of Beijing shunyi District Hospital.

### 2.2. Specimen collection

Prior to the next hemodialysis session, 5 ml of fasting peripheral venous blood was collected from each patient and sent to the laboratory within 2 hours for routine blood tests and other analyses. complete blood count was measured using the Mindray 7500 analyzer, following the instrument and reagent kit operation manuals strictly. Calculation of neutrophil-to-lymphocyte ratio (NLR), monocyte-to-lymphocyte ratio (MLR), platelet-to-lymphocyte ratio (PLR), and systemic immune-inflammation index (SII): These indices were calculated using the following formulas: NLR = neutrophil count/ lymphocyte count, MLR = monocyte count/ lymphocyte count, PLR = platelet count/ lymphocyte count, SII = platelet count × neutrophil count/ lymphocyte count.

### 2.3. Statistical analysis

Data analysis was performed using SPSS 19.0 (Chicago) statistical software and MedCalc software, and GraphPad Prism software was used for graphing. The Kolmogorov–Smirnov (K–S) test was applied to assess the normality of continuous variables, including the 4 inflammatory markers (NLR, MLR, PLR, SII). For all variables in the 3 groups (healthy control group, MHD non-anemia group, MHD renal anemia group), the K-S test results showed that the *P*-values were < .05. Therefore, differences in inflammatory markers between different groups were analyzed using the Mann–Whitney *U* nonparametric test, with *P* < .05 considered statistically significant. And apply Bonferroni correction to all statistical tests involving multiple comparisons. Pearson correlation analysis was employed to explore the relationship between novel inflammatory markers and hemoglobin levels, with a *P*-value < .05 considered statistically significant. The diagnostic performance of each inflammatory marker was evaluated using receiver operating characteristic (ROC) curve analysis, and the area under the curve (AUC) was calculated. We used the BCa bootstrap method (using MedCalc software) to verify the stability of the cutoff values. A *P*-value < .05 was considered statistically significant.

## 3. Results

### 3.1. Comparison of NLR, MLR, PLR, and SII levels among groups

**Figure 1.**
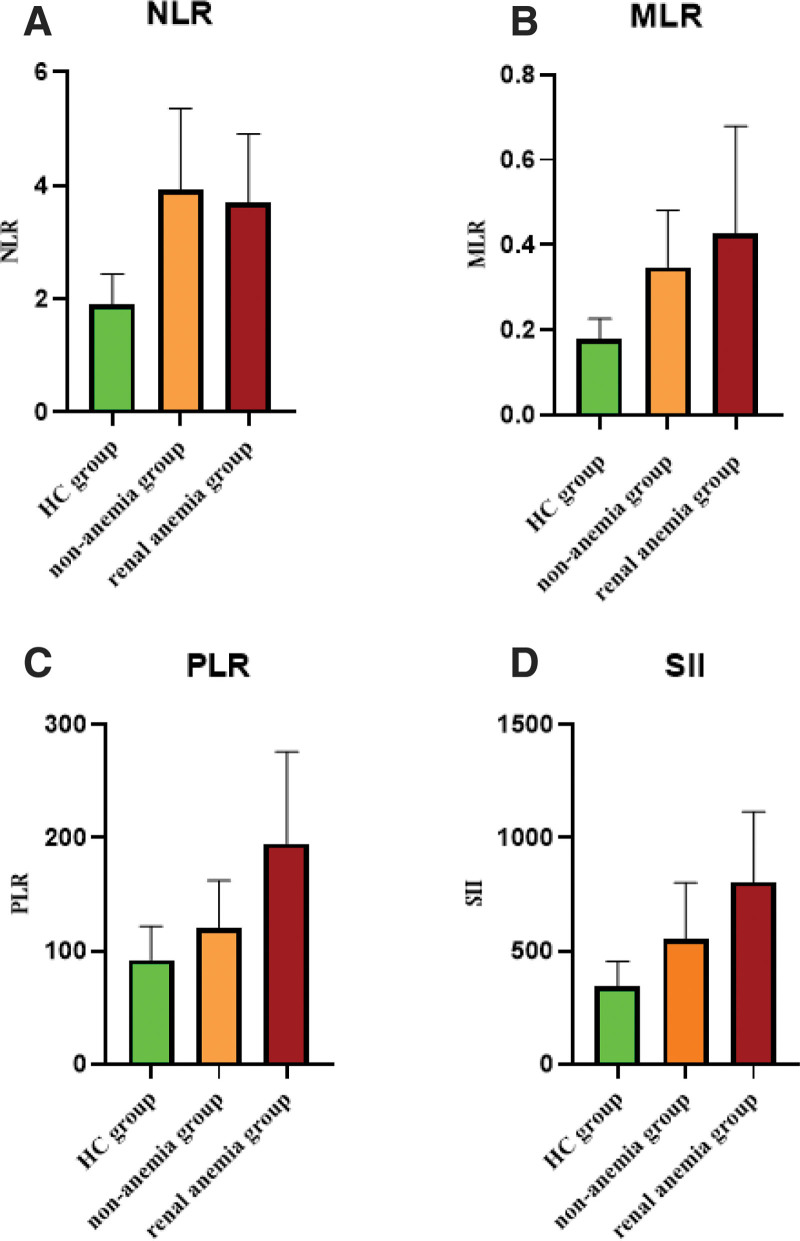
Comparison of NLR, MLR, PLR, and SII levels among the HC group, maintenance hemodialysis non-anemia group (non-anemia group), and maintenance hemodialysis renal anemia group (renal anemia group). Notes: (A) comparison of NLR levels among the 3 groups; (B) comparison of MLR levels among the 3 groups; (C) comparison of PLR levels among the 3 groups; (D) comparison of SII levels among the 3 groups. Statistical analysis was performed using the Mann–Whitney *U* nonparametric test. Exact *P*-values for pairwise comparisons are shown in Table 1. *Indicates *P* < .05, which is statistically significant. HC = healthy control, MLR = monocyte-to-lymphocyte ratio, NLR = neutrophil-to-lymphocyte ratio, PLR = platelet-to-lymphocyte ratio, SII = systemic immune-inflammation.

The levels of NLR, MLR, PLR, and SII in the healthy control group, the maintenance hemodialysis renal anemia group, and the non-anemia group are shown in Figure 1. Analysis using the Mann–Whitney *U* nonparametric test revealed that the levels of NLR, MLR, PLR, and SII in both the maintenance hemodialysis renal anemia group and the non-anemia group were higher than those in the healthy control group. Additionally, the levels of PLR and SII in the maintenance hemodialysis renal anemia group were higher than those in the non-anemia group. All these differences were statistically significant (see Table 1, *P* < .05).

**Table 1:** Comparison of NLR, MLR, PLR, and SII levels among groups (median, interquartile range) and *P*-values for pairwise comparison among groups using the Mann–Whitney *U* test, apply Bonferroni correction to adjust the *P*-value.

Inflammatory index	Level (M [Q1, Q3])			Pairwise comparison *P*-value		
	Healthy control group	MHD non-anemia group	MHD renal anemia group	Renal anemia group vs non-anemia group	Non-anemia group vs healthy control group	Renal anemia group vs healthy control group
NLR	1.85 [1.48–2.19]	3.80 [2.71–4.59]	3.48 [2.86–4.48]	1.000	<.0001*	<.0001*
MLR	0.17 [0.14–0.20]	0.32 [0.25–0.44]	0.34 [0.26–0.47]	.878	<.0001*	<.0001*
PLR	90.27 [64.69–115.51]	117.64 [92.85–136.84]	172.00 [144.94–229.68]	<.0001*	.001*	<.0001*
SII	356.98 [236.18–430.56]	515.22 [365.64–691.31]	790.83 [576.85–984.08]	<.0001*	<.0001*	<.0001*

M = medians, MHD = maintenance hemodialysis, MLR = monocyte-to-lymphocyte ratio, NLR = neutrophil-to-lymphocyte ratio, PLR = platelet-to-lymphocyte ratio, Q1 = lower quartile, Q3 = upper quartile, SII = systemic immune-inflammation.

* means *P*<.05, and the difference is statistically significant.

### 3.2. Relationship between novel inflammatory markers NLR, MLR, PLR, SII and anemia-related indicator hemoglobin in MHD patients

The correlation between novel inflammatory markers and Hb in MHD patients was explored using Pearson correlation analysis. The results showed that NLR, MLR, PLR, and SII were all negatively correlated with Hb (*P* < .05), and these differences were statistically significant (see Table 2, *P* < .05).

**Table 2:** Correlation between novel inflammatory markers and hemoglobin.

Inflammatory index	*r* value	95% Cl	*P*-value
NLR	−0.445	(−0.546 to −0.331)	<.0001*
MLR	−0.452	(−0.552 to −0.339)	<.0001*
PLR	−0.547	(−0.634 to −0.446)	<.0001*
SII	−0.531	(−0.621 to −0.428)	<.0001*

CI = confidence interval, MLR = monocyte-to-lymphocyte ratio, NLR = neutrophil-to-lymphocyte ratio, PLR = platelet-to-lymphocyte ratio, SII = systemic immune-inflammation.

* means *P* < .05, and the difference is statistically significant.

### 3.3. Diagnostic value of novel inflammatory markers NLR, MLR, PLR, SII for anemia in MHD patients

**Figure 2.**
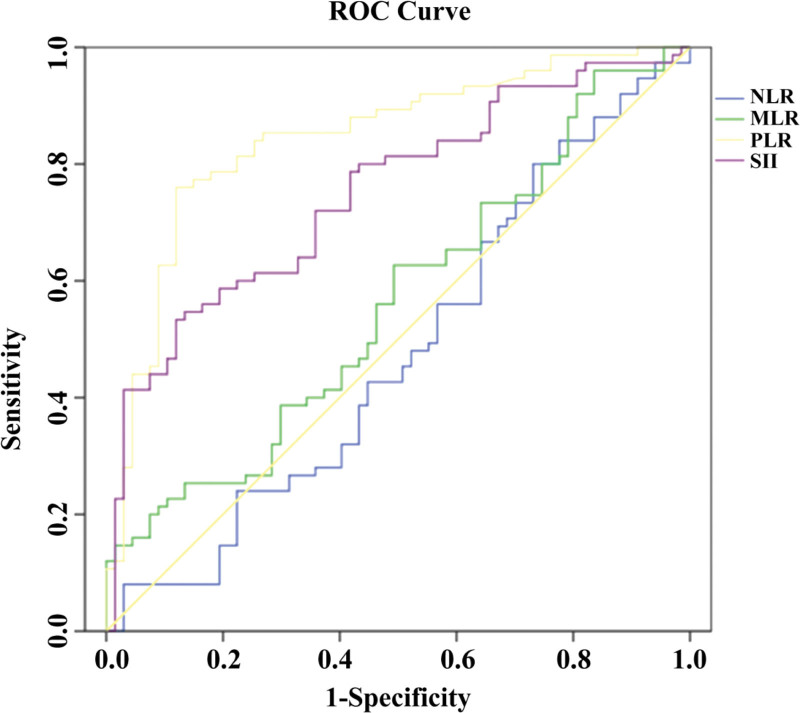
ROC curves of novel inflammatory markers (NLR, MLR, PLR, SII) for diagnosing renal anemia in MHD patients. Notes: detailed parameters (AUC, 95% CI, cutoff value, sensitivity, specificity, and *P*-value) are shown in Table 3. *Indicates *P* < .05, which is statistically significant. AUC = area under the curve, CI = confidence interval, MHD = maintenance hemodialysis, MLR = monocyte-to-lymphocyte ratio, NLR = neutrophil-to-lymphocyte ratio, PLR = platelet-to-lymphocyte ratio, ROC = receiver operating characteristic, SII = systemic immune-inflammation.

In this study, the diagnostic value of NLR, MLR, PLR, and SII for renal anemia in MHD patients was evaluated using ROC curves. The results (see Fig. 2) showed that NLR and MLR had no diagnostic value for the occurrence of renal anemia in MHD patients (*P* > .05). For NLR: The AUC was 0.524 (95% CI: 0.438–0.608), with a sensitivity of 72.0%and specificity of 40.3% at the optimal cutoff value of 4.128 (*P* = .632). For MLR: The AUC was 0.562 (95% CI: 0.476–0.645), with a sensitivity of 62.7% and specificity of 50.7% at the optimal cutoff value of 0.318 (*P* = .203). However, PLR and SII had certain diagnostic value for the occurrence of renal anemia in MHD patients. Among them, PLR had the highest diagnostic value for anemia in MHD patients, with an area under the ROC curve (AUC) of 0.844. The 95% confidence interval was 0.774 to 0.899, and the 95% Bootstrap confidence interval obtained by the BCa bootstrap method (1000 iterations, random number seed 978) was 0.770 to 0.898. The optimal cutoff value for PLR is 143.4, with a sensitivity of 76.0% and a specificity of 85.1%. The 95% Bootstrap confidence interval obtained through the BCa Bootstrap method (1000 iterations, random number seed 978) is 139.1 to 155.5, which strongly proves the reliability of PLR cutoff point diagnosis for renal anemia (see Table 3).

**Table 3:** ROC curve parameters of novel inflammatory markers for the diagnosis of renal anemia in MHD patients.

Inflammatory index	AUC	95% CI	cutoff value	Sensitivity (%) (95% CI)	Specificity (%) (95% CI)	*P*-value
NLR	0.524	0.438–0.608	4.128	72.0 (58.2–78.6)	40.3 (29.4–52.3)	.632
MLR	0.562	0.476–0.645	0.318	62.7 (51.4–72.7)	50.7 (37.6–60.9)	.203
PLR	0.844	0.774–0.899	143.4	76.0 (65.2–84.3)	85.1 (74.7–91.6)	<.001*
SII	0.750	0.671–0.819	788.1	53.3 (42.2–64.2)	88.1 (78.2–93.8)	<.001*

AUC = area under the curve, CI = confidence interval, MHD = maintenance hemodialysis, MLR = monocyte-to-lymphocyte ratio, NLR = neutrophil-to-lymphocyte ratio, PLR = platelet-to-lymphocyte ratio, ROC = receiver operating characteristic, SII = systemic immune-inflammation.

* means *P*<.05, and the difference is statistically significant.

## 4. Discussion

This study found that there were significant differences in the levels of NLR, MLR, PLR and SII between maintenance hemodialysis (MHD) patients (renal anemia group and non-anemia group) and healthy controls, and there were also significant differences in the levels of PLR and SII between renal anemia group and non-anemia group. This result is consistent with previous studies, indicating that inflammatory state is widespread in MHD patients and is closely related to the occurrence of renal anemia. For example, Atkinson MA et al pointed out that inflammation is one of the important pathophysiological mechanisms of renal anemia in MHD patients, and the increase of inflammatory indexes is positively correlated with the severity of anemia.^[15]^ In addition, Kelly DM et al also emphasized the important role of inflammation on anemia in CKD patients, which further supported the results of this study.^[16]^ In terms of mechanism, we believe that this change may be due to: the accumulation of uremic toxins activates the monocyte-macrophage system; Chronic inflammatory reaction induced by biological incompatibility of dialysis membrane;^[17]^ The vicious circle of iron metabolism disorder and inflammation.^[18]^ In particular, the significant increase of PLR suggests that platelet activation may participate in the occurrence and development of anemia by releasing pro-inflammatory factors (such as PF4 and TGF-β).^[19]^

Pearson correlation analysis showed that NLR, MLR, PLR and SII were negatively correlated with Hb (*P* < .05), which suggested that the increase of inflammation index might indicate the decrease of Hb level, that is, the more serious the inflammation, the more severe the anemia might be. Our results supported the “inflammation-anemia” axis theory proposed by Weiss et al.^[20]^ Previous studies have mostly focused on the relationship between traditional inflammatory markers such as C-reactive protein (CRP) and interleukin-6 (IL-6) and renal anemia.^[21]^ Although these traditional indicators can reflect the inflammatory state to some extent, they only represent a single inflammatory factor, and it is difficult to fully reflect the complex inflammation and immune network of the body. In this study, a number of new inflammatory indicators were innovatively introduced, and the correlation between them and Hb was clarified through quantitative analysis, especially the strong correlation between PLR and Hb, which provided a brand-new and effective tool for accurately evaluating the degree of renal anemia in clinic. In addition, the strong correlation between PLR and Hb suggests that “platelet-lymphocyte axis” may be the core regulatory pathway of MHD anemia.

In terms of diagnostic value, previous studies mostly focused on the predictive effects of NLR and PLR on cardiovascular events or all-cause mortality in patients with CKD.^[22]^ This study systematically compared the diagnostic values of NLR, MLR, PLR and SII in patients with MHD for the first time. The results showed that PLR and SII had diagnostic value for renal anemia in MHD patients, and the AUC of PLR and SII were 0.84 and 0.75, respectively, which showed high diagnostic efficiency, suggesting that PLR and SII could be used as screening tools for anemia in MHD patients. NLR and MLR cannot reliably distinguish between anemic and non-anemic patients. Turkmen K also reported a similar conclusion, stating that the PLR is a more reliable indicator of inflammation in patients with end-stage kidney disease compared to the NLR.^[23]^ Taymez et al reported a positive correlation between PLR and EHRI (indicating higher PLR = greater EPO resistance), while our Pearson correlation analysis quantifies a strong negative correlation between PLR and Hb (r = -0.547, *P* < .0001), directly linking elevated PLR to more severe anemia.^[24]^ Taymez et al primary goal was to identify whether PLR could predict EPO resistance (a specific barrier to anemia treatment). In contrast, our study directly targets the diagnostic challenge of renal anemia – a highly prevalent complication (affecting up to 90% of MHD patients) that lacks low-cost, easily accessible screening tools. We explicitly evaluated PLR’s ability to distinguish between MHD patients with and without renal anemia, filling a gap between “predicting treatment resistance” and “identifying anemia itself.”

The results of this study suggest that clinicians can use PLR as the first choice index for screening renal anemia, combined with SII for auxiliary diagnosis. From the point of clinical application, PLR and SII have significant practical advantages compared with traditional inflammatory markers CRP and IL-6. First of all, in terms of testing cost, the calculation of PLR and SII only needs to be based on the results of routine cell counts such as neutrophils, lymphocytes, monocytes and platelets in routine blood tests, and there is no need to carry out special immunological testing items, which greatly reduces the testing cost; The detection of CRP and IL-6 usually requires specific immunological detection methods (such as immunoturbidimetry, enzyme-linked immunosorbent assay, etc), which not only has high detection cost, but also requires special detection instruments and professional operators. Secondly, in terms of detection convenience, because blood routine examination is a routine monitoring item for MHD patients, PLR and SII can be monitored continuously conveniently, which is beneficial to evaluate the treatment effect and disease progress. In addition, CRP is easily disturbed by metabolic factors such as obesity and hyperlipidemia, resulting in poor specificity; IL-6 has a short half-life and the blood concentration fluctuates greatly, so it is not easy to grasp the timing of detection. In contrast, PLR and SII are less affected by these factors and have higher stability. Finally, for the comprehensive evaluation value, PLR and SII integrate a variety of blood cell information, especially SII, and contain 3 parameters of neutrophils, platelets and lymphocytes, which can reflect the inflammatory state of the body more comprehensively than a single inflammatory index.

Therefore, the application of PLR and SII as low-cost and easily available inflammatory markers in clinic will help to establish a more efficient and economical early screening system for renal anemia. According to the results of this study, it is suggested that PLR ≥ 143.4 (according to the best cutoff value of ROC) should be used as the screening threshold of anemia risk in MHD patients, and Hb should be monitored first in patients with high PLR and EPO dosage should be optimized. However, this cutoff is derived from the single-center cohort, framed as exploratory rather than clinically actionable, and requires validation in multi-center studies to confirm applicability across diverse MHD populations before potential clinical use. For patients with significantly increased PLR/SII, anti-inflammatory therapy should be considered, which may improve the curative effect of anemia. In the future, we can explore the relationship between PLR and cardiovascular events and infection risk of MHD patients, and build a multi-dimensional prognosis model.

While our study demonstrated that PLR are closely associated with renal anemia in MHD patients, we acknowledge that 3 key confounding factors – iron status, infection, and dialysis adequacy – may affect the observed results. Iron deficiency is a well-established contributor to renal anemia in MHD patients, as iron is an essential cofactor for erythropoiesis. Critically, iron metabolism is tightly intertwined with inflammation: pro-inflammatory cytokines (e.g., IL-6) upregulate hepcidin, which sequesters iron in macrophages and reduces intestinal iron absorption – creating a bidirectional “inflammation-iron deficiency” cycle.^[25]^ Uncontrolled iron deficiency could independently lower Hb levels, while concurrent inflammation (reflected by elevated PLR) might exacerbate iron deficiency. In this scenario, the observed negative correlation between PLR and Hb could partially stem from iron deficiency (not solely inflammation-driven anemia). To minimize this confounder, we excluded patients with “insufficient erythropoietin dosage” (Section 2.1, Exclusion criterion 6). Acute or chronic infection is a major trigger of systemic inflammation, which can simultaneously elevate PLR (via neutrophilia and lymphopenia) and exacerbate renal anemia (via hepcidin upregulation, impaired EPO production, and shortened red blood cell survival).^[26]^ This creates a potential confounder: infection could independently drive both elevated PLR and reduced Hb, mimicking a direct association between PLR and anemia. Therefore, patients with “severe infections” (Section 2.1, Exclusion criterion 4) were excluded and Healthy controls with “recent acute or chronic infections” (Section 2.1) were excluded to ensure the baseline inflammatory state was not skewed by infection. Inadequate dialysis leads to accumulation of uremic toxins (e.g., urea, indoxyl sulfate), which activate the monocyte-macrophage system and amplify systemic inflammation.^[27]^ This inflammation can both elevate PLR and worsen renal anemia. We ensured uniform dialysis adequacy by: Exclusion of inadequate dialysis: Patients with “inadequate dialysis” (Section 2.1, Exclusion criterion 6) were excluded. Standardized dialysis protocol: All included patients underwent consistent hemodialysis (2–3 sessions/week, 4 hours/session) using high-flux dialyzers and bicarbonate-based dialysate, ensuring reliable toxin clearance.

Through strict exclusion criteria (e.g., excluding patients with infection, inadequate dialysis, or suboptimal EPO) and standardized clinical management (e.g., uniform dialysis protocols, guideline-based iron supplementation), we minimized the impact of iron status, infection, and dialysis adequacy on our results. These measures strengthen the validity of our conclusion that PLR is independently associated with renal anemia in MHD patients. However, we acknowledge that residual confounding (e.g., mild, undiagnosed inflammation or transient iron fluctuations) cannot be fully eliminated. Future prospective, multi-center studies with serial measurements of iron parameters (hepcidin, transferrin saturation), infection biomarkers (procalcitonin), and dialysis adequacy (monthly Kt/V) could further validate our findings.

## 5. Conclusion

In summary, this study systematically compared the diagnostic efficacy of NLR, MLR, PLR and SII in MHD renal anemia for the first time, made clear the clinical priority of PLR, and provided direct evidence for simplifying the anemia screening process. Through the strong correlation between PLR and Hb, it is suggested that “platelet-lymphocyte axis” may be the core inflammatory regulation pathway of MHD anemia, which provides a theoretical basis for targeted intervention (such as antiplatelet drugs or immunomodulators). PLR and SII, as low-cost indicators derived from blood routine, can replace expensive inflammatory factors (such as IL-6 and CRP), especially in places with limited medical resources. However, our study has some limitations as it is a single-center study, with all 142 maintenance hemodialysis (MHD) patients and 74 healthy controls from the same hospital, resulting in homogeneous demographic, clinical protocol, and patient characteristics data that differ from those of different external populations, thus limiting generalizability. In addition, this study employed a cross-sectional design that captured only single time point snapshots of variables such as PLR levels and hemoglobin (Hb) levels. This design allows us to confirm the association between PLR and renal anemia, but cannot establish a causal relationship. Moreover, we lack of statistical adjustment on the medication regimen of complications (such as diabetes and hypertension) and EPO, which may introduce residual confusion and affect the reliability of the results. Therefore, long-term multi-center and large-sample studies are still needed in the future to further verify the results of this study.

## Acknowledgments

We sincerely thank the participants who volunteered for this study and the medical staff at the dialysis center and clinical laboratory of Beijing Shunyi District Hospital for their support in specimen collection and data recording.

## Author contributions

**Conceptualization:** Tingting Yang, Tianqing Cao.

**Data curation:** Tingting Yang, Jiling Zhang.

**Formal analysis:** Tingting Yang, Chengeng Liu.

**Funding acquisition:** Chengeng Liu.

**Investigation:** Tingting Yang, Chengeng Liu.

**Methodology:** Tingting Yang, Tianqing Cao, Chengeng Liu.

**Project administration:** Tingting Yang, Jiling Zhang, Chengeng Liu.

**Resources:** Tingting Yang, Jiling Zhang, Tianqing Cao.

**Software:** Jiling Zhang.

**Supervision:** Jiling Zhang.

**Validation:** Jiling Zhang, Tianqing Cao.

**Visualization:** Jiling Zhang.

**Writing – original draft:** Tingting Yang, Tianqing Cao.

**Writing – review & editing:** Tingting Yang, Jiling Zhang, Tianqing Cao.
